# The effects of hypothermia on dexmedetomidine induced contraction on human internal mammarian artery and saphenous vein

**DOI:** 10.1186/1749-8090-8-S1-O262

**Published:** 2013-09-11

**Authors:** B Oc, O Arun, S Bagcı Taylan, M Oc, H Bariskaner, A Duman

**Affiliations:** 1 Selcuk University, Department of Anesthesiology and Reanimation, Konya, Turkey; 2 Selcuk University, Department of Pharmacology, Konya, Turkey; 3 Selcuk University, Department of Cardiovasular Surgery, Konya, Turkey

## Background

Dexmedetomidine is a α2 agonist, which is used for sedation during and after surgery in patients undergoing CABG. The in vitro effects of moderate hypothermia on dexmedetomidine induced contraction on human internal mammarian arteries (IMA) and saphenous veins (SV) were studied.

## Methods

The Ethics Committee approved the study protocol. The contractile response of human IMA (n=6) and SV strips (n=6) with and without endothelium, suspended in organ baths, bubbled with 95% O2 + 5% CO2 which were subjected to cumulative concentrations of 10-9 – 10-6 M dexmedetomidine were recorded at 37ºC and at 28ºC. The results were expressed as percentage of maximum contraction to phenylephrine. Statistical Analysis: Contractions induced by dexmedetomidine were expressed as a percentage of phenylephrine induced response. Results are expressed as means ± SD. Student’t test was used for analysis between in groups and results between these groups were determined by unpaired test. A p value of < 0.05 was considered significant.

## Results

Dexmedetomidine resulted in contraction in both IMA and SV strips, in vitro. The contractions to 10-6M dexmedetomidine = IMA: 37ºC, E+: 105%, E-: 96% and 28ºC E+: 98%, E-: 97%; SV: 37ºC, E+: 85%, E-: 81% and 28ºC E+: 91%, E-: 87%. At 37ºC, dexmedetomidine caused significantly greater contraction in IMA compared to SV in strips both with and without endothelium. At 28ºC the contraction was similar in IMA and SV strips with endothelium but significantly greater in IMA strips without endothelium.

## Conclusion

Dexmedetomidine causes in vitro contraction in IMA and SV grafts. These contractions are greater in IMA compared SV strips. Endothelium derived pathways are possibly involved in the contractile responses. Hypothermia affects the mechanisms of contraction.

